# Cutaneous Manifestations in HTLV-I Positive Blood Donors

**Published:** 2013-03

**Authors:** Mohammad Javad Yazdanpanah, Masoud Maleki, Nasaibe Joneidi, Amir Reza Khalighi, Mahmoud Reza Azarpazhooh, Mohammad Khajedaluee, Farahnaz Tehranian, Majid Shahabi, Mohammad Esmaeil Khayami, Fatemeh Livani

**Affiliations:** 1 Research centre for Cutaneous Leishmaniasis, Department of Dermatology, Faculty of Medicine, Mashhad University of Medical Sciences, Mashhad, Iran; 2 Department of Internal Medicine ,Ghaem Hospital, Faculty of Medicine, Mashhad University of Medical Sciences, Mashhad, Iran; 3 Department of Neurology, Ghaem Hospital, Faculty of Medicine, Mashhad University of Medical Sciences, Mashhad, Iran; 4 Department of Social Medicine, Faculty of Medicine, Mashhad University of Medical Sciences, Mashhad, Iran; 5 Research Centre of Iranian Blood Transfusion Organization, Khorasan Razavi, Mashhad, Iran

**Keywords:** Blood donors, Dermatologic manifestation, Human T-cell lymphotrophic virus type-I

## Abstract

***Objective(s):*** Infection with the human T-cell lymphotrophic virus type-I (HTLV-I) is endemic in Mashhad, Iran. In our research we evaluated the relation between exposure to this infection and the occurrence of dermatologic manifestations.

***Materials and Methods:*** 100 blood donors, who were seropositive but asymptomatic for infection with HTLV-I, were selected as case group. They were identified by the Blood Transfusion Organization Mashhad via the ELISA test and documented by PCR. Another 100 blood donors, that were seronegative for HTLV-I via the ELISA test and who were matched to the case group for age, gender, and existence of systemic diseases, were considered as the controls. Dermatologic evaluations and skin biopsies were performed if deemed necessary, and the results were statistically analyzed.

***Results:*** 73% of the case and control groups were male, while 27% in each of these groups were female. The mean age in both groups was 40.96±11.94 years. The examination indicated that 58% of the case group and 37% of the control group had cutaneous manifestations (*P<*0.01). The most common diseases found in the case group were aphthous stomatitis, herpes labialis, and non-genital warts, while common diseases found in the control group were herpes labialis, aphthous stomatitis, and skin tag. The frequency of aphthous stomatitis, eczema, and non-genital warts in the case group were significantly more than the control group (*P<*0.05).

***Conclusion*** ***:*** Cutaneous diseases can be found more frequent in asymptomatic carriers of HTLV-I than those who are HTLV-I seronegative. The aphthous stomatitis, eczema, and non-genital warts are more prevalent in those infected by HTLV-I.

## Introduction

The human T-cell lymphotrophic virus type-I(HTLV-I) belongs to the Retroviridae family, and up to now its relationship with the adult T-cell Leukemia/Lymphoma (ATLL) and Tropical Spastic Paraparesia (TSP) has been documented. The HTLV-I infection is endemic in the southwest of Japan, the Caribbean, South and Central America, and some regions of Africa (1). Mashhad, northeast of Iran, was introduced as an endemic region of HTLV-I in 1993 (2). This virus can be transferred by breast feeding, sexual contact, and blood transfusion (3, 4).

Clinical diseases that accompany HTLV-I are rare, and 3 to 5% of carriers manifest it during their life. The disease establishment risk depends on age, duration of infection, and quality of immune system of the host. Acute seroconversion does not accompany any syndrome. The time between infection and seroconversion varies between 1 to 2 months, and the time between seroconversion and disease manifestation can vary from 18 months for HAM/TSP to many decades for ATLL. There are a wide range of clinical manifestations related to HTLV-I that may be due to virus dependent cellular transformation (e.g.ATLL), or by causing disorder in the immunological state of the host indirectly (5).

To our knowledge, several studies have been conducted on diseases accompanied with HTLV-I; however, limited studies have been done on cutaneous diseases in asymptomatic carriers (6-17).

## Materials and Methods

This is a cross sectional case–control study. The HTLV-I positive blood donors were selected as the case group, and the HTLV-I negative donors were selected as the control group. Both groups were matched for age, sex, and systemic diseases. The inclusion criterion in the case group was a seropositive PCR test for HTLV-I, and for the control group was a seronegative ELISA test for HTLV-I. The exclusion criteria were identified cases of TSP and ATLL. The sample volume included 200 participants (100 in each group).

Blood donors infected with HTLV-I were identified in the Mashhad Blood Transfusion Organization by a series of ELISA tests and verified by PCR. As a routine, these patients were referred to a neurologist for TSP screening. After the study procedure was explained to them, their written consent was obtained.

Considering patients with skin problems are banned from donating blood, to prevent bias in our study, we allowed them to be part of our research. Complete skin examination was done in both groups by the same dermatologist and in cases with an indefinite diagnosis, skin biopsy was performed. Also we considered the history of recurrent diseases and infections that were not present during examination but mentioned in a checklist of findings (recurrent aphthous stomatitis, recurrent labial herpes, recurrent furunculosis, warts, fungal diseases, cutaneous leishmaniasis, pediculosis, scabies, and herpes zoster). In examining the above cases and after offering necessary explanations to individuals with a particular disease, ensuring a relatively correct description was recorded in the checklist as positive or negative. Demographic, paraclinical, and clinical observations of participants in both groups were analyzed by SPSS software version 11.5. For qualitative variables comparisons in both groups, the Chi-square test was used; and for quantitative variables comparisons (with normal distribution), the independent t test was used; and for without normal distribution data, equivalent non-parametric tests were used. In all calculations *P<*0.05 was considered as significant.

## Results

73% of the case and control groups were male, while 27% in each of these groups were female. In both groups, the age range was 18-62 years and the mean age was 40.96±11.94 years.

Based on statistical tests, participants of the control group had a significantly higher education than the case group (*P<*0.01), and white-collar jobs were significantly more in the control than the case group (*P<*0.01). The two groups were completely matched for existence of systemic diseases.

There was not any significant difference between the two groups regarding dermatologic symptoms (xerosisand pruritus) (*P* = 0.48). In examination, skin lesions were observed in 58 participants of the case group (58%) and 37 participants in the control group (37%). The statistical tests showed that in general the existence of dermatologic manifestations in HTLV-I positive individuals was significantly more than in HTLV-I negative group (*P<*0.01). Common skin diseases in the case group listed in descending order of prevalence were: recurrent aphthous stomatitis (31%), recurrent labial herpes simplex (26%), nongenital warts (22%), skin tag (16%), fungal diseases (11%), eczema (11%), cutaneous leishmaniasis (8%), and seborrheic dermatitis (8%).

The five common skin diseases in the control group listed in descending order of prevalence were: recurrent labial herpes simplex (17%), recurrent aphthous stomatitis (14%), skin tag (12%), dry skin (7%), non-genital warts (7%), and fungal disease (6%). 11% of the case group had a type of eczema and so did three individuals (3%) in the control group, showing a significant statistical difference (*P=* 0.03). In total, 31 patients (31%) of the case group and 14 patients (14%) of the control group complained of recurrent aphthous stomatitis, and statistical analysis indicated that this disease was significantly more frequent in the case group (*P *= 0.02).

22 individuals (22%) of the case group and 7 individuals of the control group (7%) were diagnosed with non-genital warts in their examination, or it was recorded in their medical history. Statistical analysis indicated that the existence or history of non-genital warts in the case group were significantly higher than in the control group (*P<*0.01).

**Table 1:** Frequency of cutaneous manifestations in case and control group

Cutaneous Manifestations	Case		Control		*P*- value
	Frequency	(%)	Frequency	(%)	
History of recurrent aphthous stomatitis	31	31	14	14	0.01
Acne vulgaris	4	4	4	4	1
Eczema (any type)	11	11	3	3	0.03
History of alopecia areata	7	7	4	4	0.36
History of fungal disease	11	11	6	6	0.21
History of pediculosis	2	2	0	0	0.15
Psoriasis	3	3	1	1	0.31
Pigmented purpura	3	3	1	1	0.31
Tinea versicolor	5	5	1	1	0.10
Xerosis	7	7	7	7	1
Seborrheic dermatitis	8	8	3	3	0.12
History of herpes zoster	3	3	3	3	1
History of leishmaniosis	8	8	5	5	0.40
Fish tank granuloma	1	1	0	0	0.32
Recurrent furunculosis	2	2	2	2	1
Folliculitis	3	3	1	1	0.31
Multiple seborrheic keratosis	3	3	3	3	1
History of scabies	0	0	1	1	0.32
Lichen planus	1	1	1	1	1
History of non genital wart	22	22	7	7	0.01
History of drug reaction	3	3	2	2	0.64
Vitiligo	2	2	0	0	0.15
History of recurrent labial herpes	26	26	17	17	0.12
Cherry red angioma	7	7	5	5	0.39
Skin tag	16	16	12	12	0.31

**In all calculations** ***P<*** **0.05 was considered as statistically significant**

## Discussion

The relationship of HTLV-I to the adult T-cell Leukemia/Lymphoma (ATLL) and Tropical Spastic Paraparesia (TSP) has been documented (1) However, regarding skin diseases that occasionally accompany HTLV-I, there are some conflicting opinions. This is the first study on the dermatologic manifestations of asymptomatic HTLV-I positive individuals in Iran. Moreover, this study endeavors to show a clear image of this virus relationship with various skin diseases and compare its findings with the results of researchers of other endemic regions.

The demographic analysis of this study indicates that considering the level of educations and jobs, individuals in the case group belonged to a lower socioeconomic class compared to the control group. Different studies conducted in other endemic regions verify this finding (1, 18, 19). The study conducted in Brazil, (6) which had a similar number of participants to our study with 128 individuals in the case group and 108 in the control group, indicated that the most common dermatological manifestations in the case group with significant statistical differences in the order of prevalence were dermatophytosis, seborrheic dermatitis, and acquired ichthyosis; while in our study, the common diseases found in our case group in the order of prevalence were recurrent aphthous stomatitis, recurrent labial herpes simplex, non-genital warts, skin tag, fungal disease, eczema, cutaneous leishmaniasis, and seborrheic dermatitis. Among these, only recurrent aphthous stomatitis, eczema, and non-genital warts were significantly more than in the control group. This difference in results can be related to race difference, regional epidemiology of the diseases, and study methods.

The research conducted in 2005 by Lewkowicz *et al* indicated that those who had recurrent aphthous stomatitis had lower levels of CD4^+^CD25^+high^ T regulatory cells than the control group (20). Since HTLV-I has a high tendency to infect CD4^+^CD25^+high^ T regulatory cells, there is a possibility that the changes in the function of these cells are due to this infection and this justifies the prevalence of recurrent aphthous stomatitis in HTLV-I positive individuals. Some other studies indicated lower level of FOX-P3 in both CTCL (Cutaneous T Cell Lymphoma) and ATLL (Adult T Cell Leukemia/ Lymphoma) with some that demonstrated CD4^+^CD25^+^dysfunction due to viral gene product-HTLV-I tax ,that is responsible for neurological manifestation of the HTLV-I patients (21-23). In a study that examined the cellular immune response in those infected with myelopathy related to HTLV-I, it was concluded that cellular immune function was significantly faulty in these patients (24).

Another article that reported a case of hyperinfection strongyloidosis in a HTLV-I carrier, proposed that the HTLV-I infection in some individuals can cause a selective immunodeficiency against strongyloidosis (25). Therefore, it is possible that the high prevalence of non-genital warts in HTLV-I positive individuals in this study also had a special immunodeficiency against the human papillomavirus, which needs further studies to prove.

The link between infective dermatitis and HTLV-I has been identified (15). Also in a study in 2002, it was determined that the rate of HTLV-I positivity in patients with nonspecific dermatitis was significantly more than the control group (26). The results of this study also indicate the link between eczema and HTLV-I, so that the existence of eczema in the case group was significantly more than the control group.

Another study indicates a higher frequency of skin diseases in cases with HTLV-I infection (27). In our study, the prevalence of skin diseases also in those who are infected with this virus was significantly higher than in non-infected individuals.

The comparison between 15 HTLV-I positive individuals with 15 HTLV-I negative individuals concerning cutaneous manifestations in Brazil indicates that dry skin is more common in the first group (8). However, in our study there was not a significant difference among the two groups regarding dry skin. Since the number of participants in our study was more, we can conclude that our results are more reliable.

In another article, due to the higher incidence of crusted scabies in HTLV-I positive individuals, this disease was considered as a marker in tests for HTLV-I in the endemic region (28). However we had not have any case of scabies in this study. 

Vitiligo along with HTLV-I has been reported (7). Another study conducted by Javidi *et al* in Mashhad, did not show any link between the virus and vitiligo (29). In our study, two individuals in the case group had vitiligo, while in the control group there was no case of this disease (*P=*0.9). Therefore, our study also did not show any association between HTVL1 and vitiligo. 

Other skin diseases mentioned that accompany HTLV-I are: dermatomyositis (7), impetigo, rosacea, acne (8), kikuchi`s disease (14), psorisiform lesions (30), and polyarteritis nodosa (10). In our study among these diseases only acne and psoriasis accompanied HTLV-I and their prevalence did not have any significant statistical difference.

Recent studies suggested that high HTLV-I pro-viral load to be a marker for HTLV-I associated with myelopathy/tropical spastic paraparesis, and it was indicated that HTLV-I proviral load is significantly higher in infective dermatitis associated with HTLV-I (IDH) than in HTLV-I carriers (31, 32). However we did not evaluate this marker in our cases, and it would be better for other investigators to determine it as a factor for relationship between various clinical presentations and HTLV-I positivity. We also recommend further studies in different endemic regions of HTLV-I with greater sample size.
